# Effectiveness of quality incentive payments in general practice (EQuIP-GP): a study protocol for a cluster-randomised trial of an outcomes-based funding model in Australian general practice to improve patient care

**DOI:** 10.1186/s12913-019-4336-2

**Published:** 2019-07-29

**Authors:** Gregory M. Peterson, Grant Russell, Jan G. Radford, Nick Zwar, Danielle Mazza, Simon Eckermann, Judy Mullan, Marijka J. Batterham, Athena Hammond, Andrew Bonney

**Affiliations:** 1 School of Medicine, University of Tasmania, Hobart and Launceston, Tasmania, Australia; 2 Department of General Practice, Monash University, Clayton, Victoria Australia; 3 Faculty of Health Sciences & Medicine, Bond University, Robina, Queensland Australia; 4 Australian Health Services Research Institute, University of Wollongong, Northfields Ave, Wollongong, NSW Australia; 5 School of Medicine, University of Wollongong and Illawarra Health and Medical Research Institute, Northfields Ave, Wollongong, NSW Australia; 6 Statistical Consulting Centre, School of Mathematics and Applied Statistics, University of Wollongong; National Institute for Applied Statistics Research Australia, University of Wollongong; and Illawarra Health and Medical Research Institute, Northfields Ave, Wollongong, NSW Australia; 7 School of Medicine, University of Wollongong, Northfields Ave, Wollongong, NSW Australia

**Keywords:** Primary care, General practice, Quality, Continuity of care, Funding, Incentivisation, Health service utilisation

## Abstract

**Background:**

There is international interest in whether improved primary care, in particular for patients with chronic or complex conditions, can lead to decreased use of health resources and whether financial incentives help achieve this goal. This trial (EQuIP-GP) will investigate whether a funding model based upon targeted, continuous quality incentive payments for Australian general practices increases relational continuity of care, and lessens health-service utilisation, for high-risk patients and children.

**Methods:**

We will use a mixed methods approach incorporating a two-arm pragmatic cluster randomised control trial with nested qualitative case studies. We aim to recruit 36 general practices from Practice-Based Research Networks (PBRN) covering urban and regional areas of Australia, randomised into intervention and control groups. Control practices will provide usual care while intervention practices will be supported to implement a new service model incorporating incentives for relational continuity and timely access to appointments. Patients will comprise three groups: older (over 65 years); 18–65 years with chronic and/or complex conditions; and those aged less than 16 years with increased risk of hospitalisation. The funding model includes financial incentives to general practitioners (GPs) for providing longer consultations, same day access and timely follow-up after hospitalisation to enrolled patients. The payments are proportional to expected health system savings associated with improved quality of GP care. An outreach facilitator will work with practices to help incorporate the incentive model into usual work. The main outcome measure is relational continuity of care (Primary Care Assessment Tool short-form survey), with secondary outcomes including health-related quality of life and health service use (hospitalisations, emergency presentations, GP and specialist services in the community, medicine prescriptions and targeted pathology and imaging ordering). Outcomes will be initially evaluated over a period of 12 months, with ongoing data collection for 5 years.

**Discussion:**

The trial will provide robust evidence on a novel approach to providing continuous incentives for improving quality of general practice care, which can be compared to block payment incentives awarded at target quality levels of pay-for-performance, both within Australia and also internationally.

**Trial registration:**

Australian New Zealand Clinical Trials Registry ACTRN12618000105246. Registered on 23 January 2018.

## Background

It is clear that health care systems with strong primary care have better population health [1], lower rates of unnecessary hospitalisations and more equitable health outcomes [2]. Key components of primary care that contribute to improved population health outcomes include providing affordable first access to the health system for all medical needs; person-(not disease) focused care over time; comprehensiveness of care; and coordination of care [1, 3]. At the same time it has become clear that primary care fee-for-service health payment systems, as in Australia, need fundamental reform to create incentives better aligned to quality of care, rather than encouraging high-volume short consultations with resulting downstream health and health system impacts in populations over time [4].

International evidence has highlighted the significant benefits that relational continuity (an ongoing therapeutic relationship with one or more providers [5]) has for quality and outcomes of primary care, including decreasing hospitalisations for Ambulatory Care-Sensitive Conditions (ACSCs) and reducing mortality risk [6–8]. There is also evidence that access to longer consultations has a range of benefits in terms of the GP-patient relationship and in the quality of assessment, dealing with complexity, care planning and preventive care [9–14]. However, under the current fee-for-service system for GPs in Australia, rebates under the Medicare Benefits Schedule (MBS), subsidised by the Federal Government, are proportionally less for time spent conducting longer encounters than shorter encounters. This feature incentivises rapid patient turnover and has the potential to contribute to lower quality of care, particularly for patients living with chronic disease [15, 16].

Consequently, with financial assistance from the Federal Government, the Royal Australian College of General Practitioners (RACGP) sought proposals to develop and trial alternative funding models to support high-quality primary care. This trial formed the basis of one of the funded proposals.

### Objectives

The study aims to evaluate the impact of a new funding model in primary care, comprising targeted incentives for enrolment with a preferred provider, longer consultations, same day access and structured follow-up after hospitalisation, on the quality of care and health service utilisation, and related costs for patients at increased risk of hospitalisation. The impact of the intervention will be compared to usual care.

The funding model trial will test incentives for specific quality improvement factors in high-risk chronic disease populations and provides payment incentives that are proportional to the expected health system cost savings resulting from those patients’ care improvement. This model enables incentives for continuous quality of care improvement within existing overall health system budgets, rather than previous quality incentive models with fixed block payments at target thresholds having localised incentives and without budgetary justification [17–19].

The primary hypothesis is that among high-risk patients and children attending general practices, the introduction of a practice-level service model incorporating continuous and graded quality improvement incentives, will improve patient-perceived relational continuity. Our specific research questions are:

- In patients over the age of 65 living with chronic illness, what is the impact of an incentivised practice-level service model promoting access to preferred primary care providers, longer consultations and structured post-hospitalisation follow-up on:
  - patient-perceived relational continuity of care;
  - referrals for specified radiology and pathology services;
  - medicine prescriptions;
  - potentially avoidable hospitalisations (PAHs); and
  - emergency department attendances.
- Does the application of this model in patients under 16 years of age (with defined comorbidities) provide improved access to same day care?
- Is the model cost-effective when used in each population?
- How acceptable is the model for patients and staff members of primary care practices?

## Methods/design

### Overview

A mixed methods approach, incorporating a two-arm pragmatic cluster randomised controlled trial (RCT) with nested qualitative case studies, will be used to investigate the effectiveness, acceptability, resource use and costs of the service model in general practice. Pragmatic RCTs have emerged as a way of bridging the gap between traditional RCTs, which have a good internal validity, and observational studies, which have good external validity [20]. Pragmatic trials measure effectiveness and seek to maximise external validity to ensure that the results can be generalised [21]. To decrease contamination bias we will use cluster randomisation at the general practice level, where practices (and their associated physicians), rather than the individual patients, are randomised to the intervention or usual care arm funding models [21].

Our theoretical framework for the study is informed by the literature on organisational change, where organisations are considered as a whole, rather than as a set of independent attributes [22–25], and methods for structuring continuous quality of care improvement incentives [17–19]. Three principles underpin the work.

- Use of the United Kingdom Medical Research Council framework for designing complex interventions, where previous data from baseline qualitative and observational work will be incorporated into the design and evaluation of a trial of a complex intervention [26].
- Implementation based on intervention facilitation in primary care, where skilled individuals work to identify and overcome challenges in implementing evidence-based care and inefficiencies in non-clinical routines and processes [27].
- A realist approach to the process evaluation of the intervention [28], seeking to identify “what worked for whom, in what context, when and why” [29].

Quality improvement research has been criticised for not capturing a sufficient understanding of the organisational change process about why interventions do or do not work [23]. Therefore, this study is also incorporating qualitative case studies to investigate barriers and enablers of the service model within the intervention general practices.

The qualitative component of the research will provide insights into the impacts of the intervention on patients and general practice staff; develop an understanding of how the intervention is experienced by participants and assess its acceptability from multiple perspectives; explore and describe the contexts, mechanisms and outcomes that influence uptake and implementation of the intervention; and investigate the attitudes and beliefs of patients and general practice staff about the use of financial incentives in the general practice setting.

### Setting

The study will be set in 36 general practices across three practice-based research networks (PBRNs) in NSW, Victoria and Tasmania (Table 1). The study’s three collaborating centres enable coverage and generalisability to practices across regional, rural and urban settings.

**Table 1:** Details of the study’s three collaborating practice-based research networks (PBRNs)

Name of Practice Based Research Network	The Northern Tasmanian Practice Based Research Network	Monash Practice Based Research Network (MONReN)	The Illawarra and Southern Practice Research Network (ISPRN)
Region	Northern Tasmania	South-East Melbourne	South-East New South Wales
Affiliated organisations	University of Tasmania	Monash University	University of Wollongong
Regional population	Northern Tasmania	Inner East Melbourne, Bayside, South Eastern, Eastern and Frankston and Mornington Peninsula regions of Melbourne	Illawarra, Shoalhaven, Southern NSW, Murrumbidgee, Bega
Number of practices in region	28	479	185
Potential PBRN eligible practices	13	290	40

### Practice inclusion criteria

Practices will:

- be delivering generalist (i.e. non-specialised) primary care medical services and employing at least one full-time equivalent GP;
- have been in business for at least 1 year and not be intending to close for a further 2 years.
- be able to generate patient encounter data through either of the two most commonly used general practice clinical software systems in Australia (Medical Director or Best Practice);
- consent to the use of NPS MedicineWise MedicineInsight clinical data extraction software [30]; and
- not be registered as participants or potential participants in the Australian Government Health Care Homes trial [31].

### Practice recruitment

**Fig. 1:**
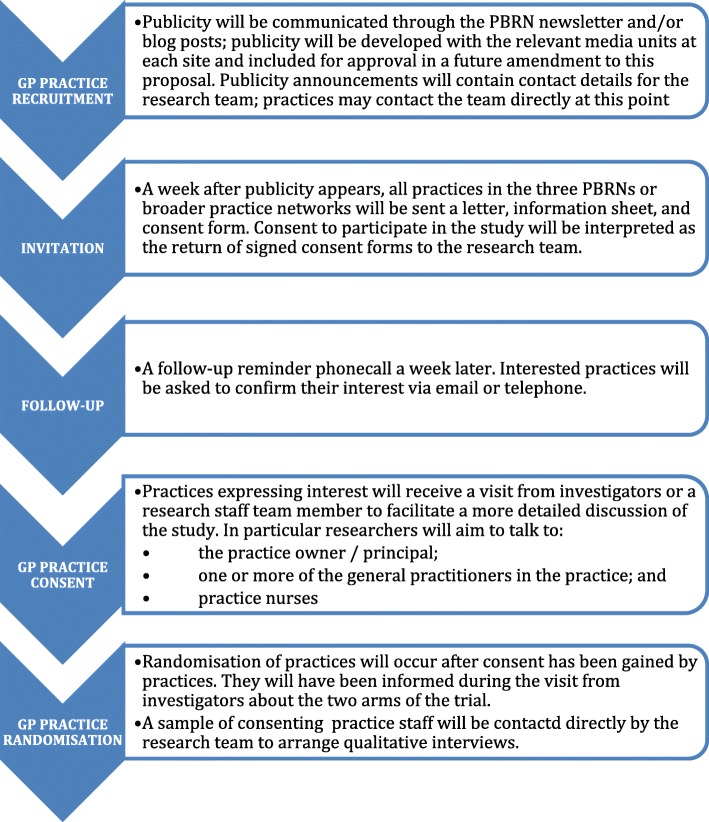
General practice recruitment approach

Recruitment will commence through the three collaborating PBRNs. Publicity will be developed with the relevant media units at each site and communicated through the PBRN newsletter and/or blog posts. Publicity announcements will contain contact details for the research team, allowing eligible practices to contact the team directly at this point. A week after publicity appears, all eligible practices in the PBRN will be sent a letter, a comprehensive information pack and consent form. The research team will contact non-responding practices by phone 1 week after the information mail-out. Practices expressing interest in participation will receive a visit from investigators or a research team member to facilitate a more detailed discussion of the study. The general practice recruitment approach is outlined in Fig. 1.

### Practitioner and support staff recruitment

Once practices have consented we will seek to identify individual general practitioners (GPs) and general practice registrars (trainees)(GPRs) under supervision, along with practice nurses (PNs) and practice manager (PMs), to participate. To reflect current work practices, GPs/GPRs with full-time or part-time work patterns will be included. GPs are not eligible if they provide a limited scope of practice (e.g. only skin cancer surgery). As part of the recruitment process, GPs, PNs and PMs who agree to participate will sign the consent forms.

### Patient inclusion criteria

Three groups of ‘high-risk’ patients will be included in the study:

- aged over 65 years;
- aged 18–65 years with common chronic and/or complex ACSC (chronic obstructive pulmonary disease (COPD), diabetes, angina (or ischaemic heart disease), cardiac failure, asthma); or
- aged less than 16 years and at risk of hospitalisation, defined by previous diagnosis with a high-risk condition (e.g. asthma, epilepsy, dental condition, acute bronchiolitis, pneumonia or croup).

### Patient recruit1ment

Following practice randomisation, in both intervention and control practices, an electronic database search will be conducted by the PN, PM or appropriate administrative staff member (assisted by a research team member, when necessary) using the practice’s own clinical software search facility to identify active patients (attended the practice three or more times in the last 2 years) who meet the inclusion criteria for the study.

Practices will generate lists of eligible patients seen by participating GPs, using 31st May 2018 as an index consultation day from which to extend searches. The generated lists will be screened by patients’ GPs to exclude patients unable to understand English or with significant cognitive impairment or distress. Then, the practice will post out a supplied information pack, consisting of a letter of invitation, an information sheet and a consent form, to a total of 200 eligible patients. This process will be the same in both the intervention and control groups. Information packs will be distributed to a sample of three patient cohorts from each practice, as follows:

- *n* = 60 patients aged over 65 years;
- n = 60 patients aged 18–65 years with chronic and/or complex ACSCs; and
- *n* = 80 parents of eligible children aged less than 16 years.

From the 200 information packs it is anticipated that, via an expected response rate of 25%, we can meet the target recruitment of 50 patients per practice. If recruitment is less than anticipated, a further wave of invitations will be sent and/or GPs can direct eligible patients to the trial information packs in each surgery. Patients’ consent to participate will be interpreted as the return of a completed consent form to the practice/the research team.

### Randomisation of practices

We will use randomisation by minimisation to reduce the risk of the trial being unbalanced across arms by practice size (less than or equal to 5 full-time equivalent GPs, or 6 or more) and Index of Relative Socioeconomic Disadvantage (IRSD) at each practice’s local government area (whether in the most disadvantaged 40% or not, using Socio-Economic Indexes for Areas (SEIFA) [32]. Randomisation will be dynamic and conducted by the trial statistician (MJB) as practice level consent is obtained. The identity of the practices will be masked from the statistician until after the analyses are performed. The project officers in each state will enter a code for each consented practice into a cloud-based database in the order that the practice consents are received and notify the statistician by email. The statistician will then perform dynamic randomisation by minimisation, balancing for practice size and IRSD, entering the allocation into the database. With the exception of the statistician, all participants and project staff will be aware of the practice allocation to the control or intervention arms of the trial.

### Intervention

Intervention general practices will be supported to implement a new service model incorporating quality incentives for patients’ relational continuity and timely access. The intervention itself will comprise a mechanism of, firstly, patient enrolment within a practice. The practice will then offer enrolled patients: a) guaranteed access to a minimum of three longer appointment types over the study period for older patients or those with chronic or complex illness; b) a review visit within 7 days of admission to an emergency department or hospital, or after other significant health events; and c) for patients aged less than 16 years, same day access for acute conditions.

**Fig. 2:**
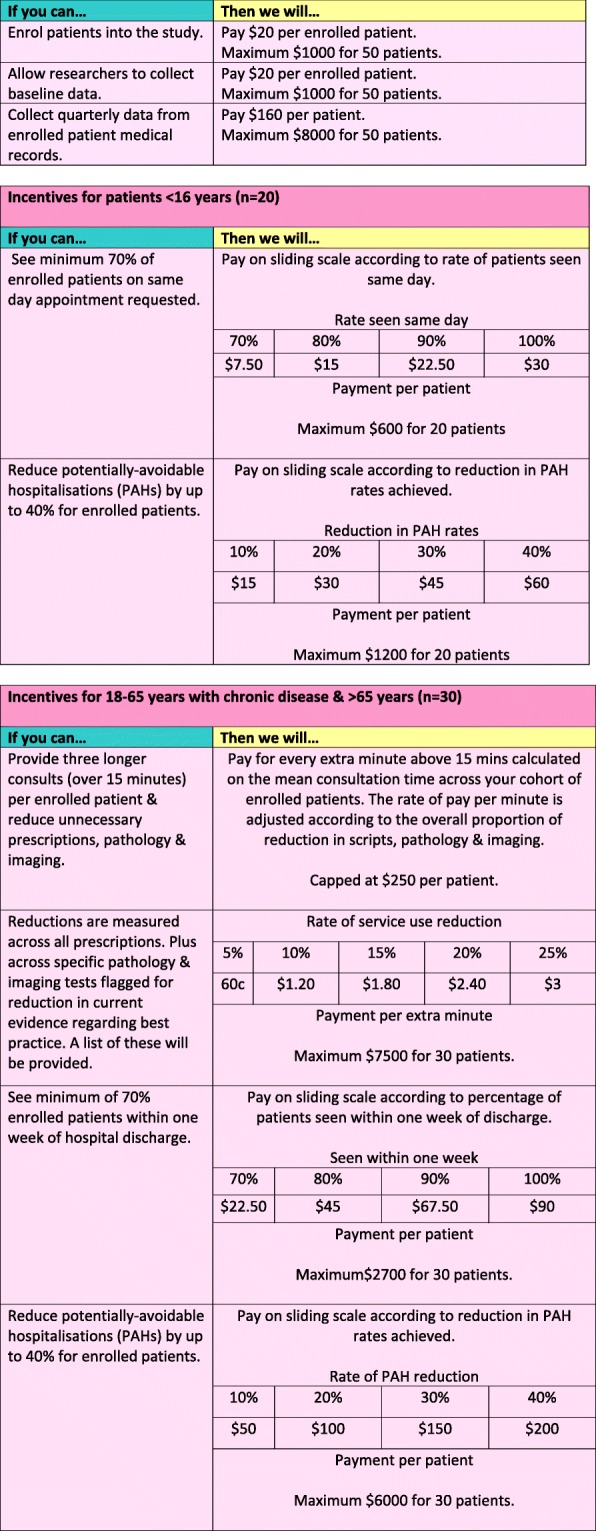
Plain language incentive structure (as provided to participating general practices)

Figure 2 summarises the incentive structure. All incentives are calculated over the 12 months of the trial and compared with the 12-month period immediately previous to the trial. The incentive payments apply to the percentage of change achieved across the group of enrolled patients at the level of the practice. The incentives supplement the existing payment arrangements. All incentives will be calculated and paid at the conclusion of the 12-month trial.

An intervention facilitator (IF) will be employed by each of the three collaborating centres. They will work with practices in the intervention arm of the study to facilitate implementation of the new service model. They will also collect data relevant to their facilitator role using structured diary keeping. These diaries will contribute data concerning practice culture, communication and routines, plus information about the challenges and successes of the intervention. The EQuIP-GP Trial timeline is summarised in Table 2.

**Table 2:** EQuIP-GP Trial timeline

Task Name	Start	Finish
*Practice Recruitment*	April 1 2018	November 30 2018
○ Publicity will be communicated through the PBRN newsletter and/or blog posts		
○ After publicity appears, all practices in the three PBRN’s will be sent a letter, information sheet and consent form.		
○ Practices expressing interest will receive a visit from investigators or a research staff team member to facilitate a more detailed discussion of the study.		
Practice Randomisation ○ Dynamic randomisation of practices will occur after consent has been gained by practices.		
Data collection ○ Distribution of pre-trial provider and facility surveys to practices		
○ A sample of consenting practice staff will be contacted directly by the research team to arrange qualitative interviews following randomisation		
*Patient Recruitment*	June 1 2018	December 31 2018
○ A Practice Nurse in the consenting practices will run an electronic search of the patient database in order to identify potential participants who meet the inclusion criteria		
○ A sample of 200 patients identified in the electronic search will be sent an information pack containing a letter of invitation, an information sheet and a consent form. ○ After 2 weeks, if recruitment is insufficient, then direct invitation to patients presenting to the practice will commence		
Baseline data collection ○ Distribution of pre-trial patient surveys: PCAT, EQ-5D-5 L HRQOL and ease of access survey to parents/guardians of <16 patients.		
○ A sample of consenting patients will be contacted directly by the research team to arrange qualitative interviews following recruitment.		
*Intervention period*	August 1 2018	July 31 2019
Intervention facilitation		
○ Intervention facilitators to visit intervention GP practices 3 times	August 1 2018	January 31 2019
Follow-up data collection	July 1 2019	August 31 2019
○ A sample of consenting practice staff will be contacted directly by the research team to arrange follow-up qualitative interviews.		
○ Distribution of post-trial patient surveys: PCAT, EQ-5D-5 L and ease of access survey to parents/guardians of <16 patients.		
○ A sample of consenting patients will be contacted directly by the research team to arrange qualitative interviews.		
○ Intervention facilitator interviews		
○ Electronic Health Record data extraction for period August 1 2017 – July 31 2019		

### Standard care

Control practices will provide usual care. Usual care in the context of this study is care received by patients in daily practice in regard to their particular health concerns [21], and represents normal practice against which to compare the intervention.

Both intervention and control practices will be provided access to links to quality improvement education materials (e.g. including Choosing Wisely Australia recommendations for improving the quality of healthcare by eliminating unnecessary and sometimes harmful tests, treatments, and procedures [33]).

### Discontinuation criteria

General practices and individual patients can withdraw at any time, without reason. There are no specific discontinuation criteria. Practices and patients can request withdrawal of their data if they discontinue up until the time of analyses. Unless requested by the participant, data will be retained for all participants including withdrawals and deviations from the protocol.

### Outcomes

The primary outcome of the trial is relational continuity, as measured in the appropriate scale of the 43-item shortened Primary Care Assessment Tool (PCAT) [34]. The domains of accessibility, coordination and comprehensive care in participants will also be measured using the relevant domain scales of the PCAT [34].

We will assess the use of care-related services in the 12 months prior to the trial in comparison with the 12-month trial period. Data elements include:

- total prescription counts per patient and Pharmaceutical Benefits Scheme (PBS) costs;
- selected pathology services (microscopy, culture and sensitivity testing for leg ulcer swabs or urine, full blood count, erythrocyte sedimentation rate, thyroid function tests, prostate specific antigen and serum vitamin D level) per patient and Medical Benefits Schedule (MBS) costs;
- selected radiology services (lower back and chest x-rays, carotid duplex studies, computed tomography of the head) per patient and MBS costs; and
- total specialist referrals per patient and MBS costs.

We will also assess and collect data on hospitalisation and emergency department attendance rates in the 12 months prior to the trial in comparison with the 12-month trial period and for 5 years following the trial. Due to funding and time constraints, it is not possible to adequately power the trial to measure a reduction in PAHs within the study period. Therefore, the study investigators will establish linked data collection procedures to enable ongoing data collection and analysis for up to 5 years. Patient mortality rates will also be assessed for up to 5 years.

**Fig. 3:**
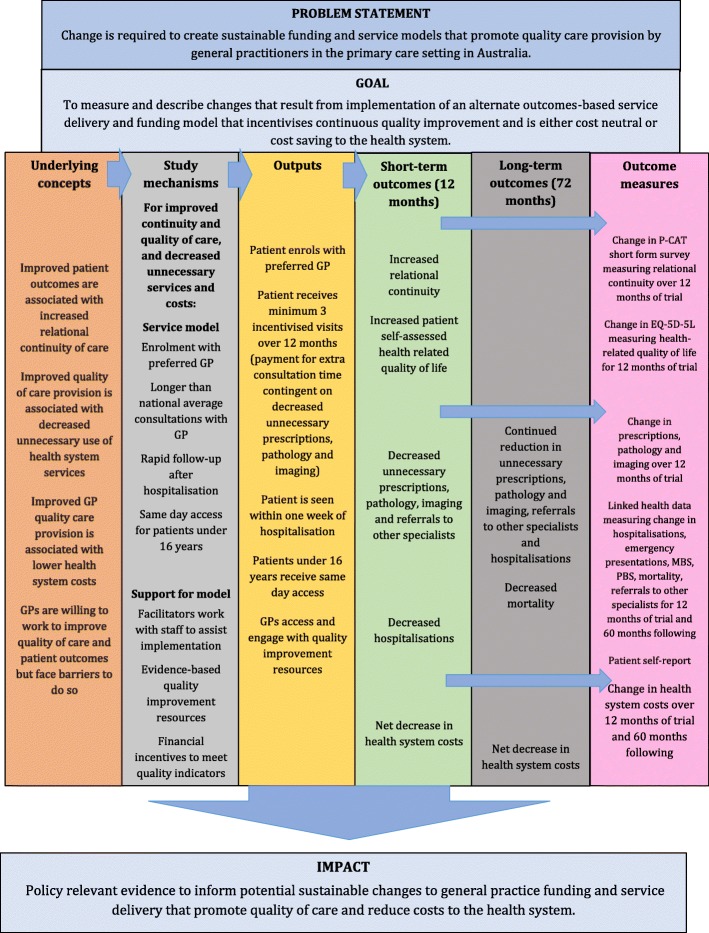
EQuIP-GP program logic

The proportion of patients seen within 1 week of discharge from hospital, and under 16-year-olds seen on the same day as requested for acute conditions, will be assessed using quarterly manual chart audits of enrolled patients in each practice performed by the PNs. In addition, patient/parent perceived ease of access will be measured by a brief survey at the beginning and conclusion of the trial, along with health-related quality of life (EQ-5D-5 L) [35]. Table 3 outlines the EQuIP-GP data variables, and Fig. 3 the program logic for the trial.

**Table 3:** EQuIP-GP data variables

Data level	Variable	Dependent (DV) or independent variable (IV)	Source
Practice	Locality IRSD	IV	Australian Bureau of Statistics [28]
	Practice size - 6 or more GP FTEs	IV	Practice
	CIHI Practice survey^a^	IV	Practice
GP	CIHI Provider survey^b^	IV	GP
Patient	EQ-5D-5 L baseline^c^	IV	Patient self-completion
	EQ-5D-5 L trial completion^c^	DV	Patient self-completion
	PCAT baseline (primary outcome measure)	IV	Patient self-completion [29]
	PCAT trial completion (primary outcome measure)	DV	Patient self-completion [29]
	Hospitalisations previous 12 months (emergency department [ED] + admission)	IV	Patient self-completion
	Hospitalisations during the trial (ED + admission)	DV	Patient self-completion
	Same day consultations <16 years previous 12 months	IV	Patient self-completion
	Same day consultations <16 years during the trial	DV	Patient self-completion
	1 week post-hospital consultations previous 12 months	IV	Patient self-completion
	1 week post-hospital consultations during the trial	DV	Patient self-completion
	DOB	IV	Electronic health record (EHR)
	Sex	IV	EHR
	Smoker	IV	EHR
	Indigenous	IV	EHR
	Diagnoses	IV	EHR
	Medication list number	IV	EHR
	Prescriptions previous 12 months	IV	EHR
	Prescriptions ordered during the trial	DV	EHR
	Pathology received previous 12 months	IV	EHR
	Pathology received during the trial	DV	EHR
	Imaging received previous 12 months	IV	EHR
	Imaging received during the trial	DV	EHR
	Chief diagnoses	IV	EHR
	Time from hospital discharge to appointment	IV	EHR
	Time from request for same day appointment (< 16 yo) to being seen	IV	EHR
	Consultation number	IV	EHR
	Consultation time length	IV	EHR
	Hospitalisations previous 12 months (ED + admission)	IV	EHR
	Hospitalisation during the trial (ED + admission)	DV	EHR
	Mortality during the trial	DV	EHR
	Hospitalisation previous 12 months (ED + admission)	IV	State hospital data
	Hospitalisation during the trial and 5 years following (ED + admission)	DV	State hospital data
	Prescriptions filled previous 12 months	IV	PBS
	Prescriptions filled during the trial and 5 years following	DV	PBS
	Pathology billed previous 12 months	IV	MBS
	Pathology billed during the trial and 5 years following	DV	MBS
	Imaging billed previous 12 months	IV	MBS
	Imaging billed during the trial and 5 years following	DV	MBS
	Specialist consultations previous 12 months	IV	MBS
	Other specialist consultations during the trial and 5 years following	DV	MBS
	Mortality during the trial and 5 years following	DV	Deaths registry

^a^Canadian Institute for Health Information (CIHI). Measuring Organizational Attributes of Primary Health Care Survey. Available at: https://www.cihi.ca/sites/default/files/info_phc_organize_en.pdf [Accessed 8 January 2019]

^b^Canadian Institute for Health Information (CIHI). Attributes of Primary Health Care: Provider Survey. Available at: https://www.cihi.ca/sites/default/files/document/info_phc_provider_en.pdf [Accessed 8 January 2019]

^C^5-level EQ-5D version (EQ-5D-5 L). Available at: https://euroqol.org/eq-5d-instruments/eq-5d-5l-about/ [Accessed 8 January 2019]

### Qualitative substudy

The aim of the qualitative component is to provide context and insight into the experience, acceptability and success of the intervention from the perspective of patients, GPs and PMs. A case study methodology will be used. It is recommended in health research when studying complex phenomena and the context in which they are embedded [36]. The methodology is useful for conducting evaluations and developing interventions, both of which are relevant for this study. The constructivist underpinnings of case study methodology privilege the existence of multiple perspectives and subjective meanings [37], making it ideal to investigate the experiences of different staff members and patients within the same practice. Here, the ‘case’ to be examined at each practice is the uptake and implementation of an intervention using financial incentives.

The qualitative component combines interviews and the IF’s diary records. Semi-structured interviews will be conducted over the telephone or may be conducted in person if requested and where logistically viable. A total of 60 interviews (24 with patients, 24 with GPs and 12 with PMs) will be conducted – 30 at baseline and 30 at the conclusion of the 12-month trial. These will be divided evenly across the three collaborating PBRNs and conducted in intervention practices only. The case study sites will be purposively sampled using SEIFA code and practice size as descriptors to ensure diversity. A maximum variation approach to sampling of patients (age, gender, comorbidities) will be applied to invitation for the qualitative interviews.

### Data management

On receipt of their consent forms, all participants (practice and patient) will be assigned a code, which will link their data to a master-sheet which will contain participants’ demographic details and location. The master-sheet will be kept secure and confidential by the project coordinator in a password-protected file in the project data management system. All data for the evaluation of the project will be identified by the participants’ code only. This is so that the data can be kept confidential, but can be re-identified to enable appropriate grouping and analysis in the case studies at the end of the study. As general practices will be involved in the extraction of their own consultation time and access data, data extraction will be performed by a staff member of the practice who has usual access to the data.

All participants (practices and patients) will have the choice of providing their data in hard copy or online data collection forms. We will use ‘CloudStor’ as a secure, password-protected, cloud-based data collection and storage facility for participant-provided project research data [38]. The CloudStor server for the project is located in Australia. Permission for access to the data within the research team will be an only-if-needed basis. Hard copies of all consent forms will be stored in locked filing cabinets in the Departments of General Practice at each respective university (or password-protected folder on CloudStor if in soft copy form).

After data integrity checks, data extracts received from MedicineInsight will be stored in CloudStor. Data collected from the MBS, PBS, hospitalisation and deaths registries will be collected for 5 years following the trial, but only with express consent from the participants. These data will be stored and analysed in the Secure Unified Research Environment (SURE) maintained by the SAX Institute [39]. SURE is a purpose-built remote-access data research laboratory and storage facility with servers located in Australia.

Interview and IF diary data will be stored securely at each site during the collection period.

De-identification will take place after transcription of interviews. The code sheets and de-identified transcripts will be stored separately in CloudStor.

### Quantitative data analysis

Baseline characteristics will be summarised at the collaborating PBRN centre and general practice levels within each trial arm, to provide counts and frequencies for categorical variables and means with standard deviations or medians and ranges for continuous variables, as appropriate. The primary outcome measure is a between-group difference in changes in the domain of relational continuity, as measured in the appropriate scale of the PCAT [34] at completion of the trial in comparison with baseline. Between-group differences in the domains of accessibility, coordination and comprehensive care in participants will also be measured and tested using the relevant domain scales of the PCAT [34]. To account for the potential effects of clustering, we will use hierarchical linear models with random main effects specified at the general practice level. Total prescription counts for the 12 months prior to trial commencement will also be used to control for case-mix across the trial.

Similarly, each of the other specified outcome variables (Table 2) will be statistically analysed using multi-level modelling methods to test for between-group (intervention vs. control) differences in the change in variables over the course of the study period. It is intended that data collection from administrative datasets (MBS, PBS and hospital admissions) will be continued beyond the trial period for 5 years, and analysed similarly. Data will not be excluded on the basis of non-adherence or protocol violation. The mixed models treat the data as missing at random and incorporate partial datasets, and are appropriate for analysis of datasets with missing data.

### Qualitative data analysis

Using a realist evaluation approach to analysis, we will iteratively generate context-mechanism-outcome configurations (CMOc) to clarify what works for whom, why, when and in what context at the individual and practice level [29]. A framework analysis approach will allow us to identify relationships and patterns across diverse data sources, including transcripts, documents, diary entries and observations. Framework analysis has been widely used in health research [40, 41]. Importantly, when using nested case studies as we are, framework analysis facilitates an ordered and organised way to compare and contrast data both across multiple cases and within individual cases.

We will use the seven-step process proposed by Gale et al. [42] to undertake the framework analysis. These steps are 1) transcription; 2) familiarisation; 3) coding; 4) developing a working analytical framework; 5) applying the framework; 6) charting the data; and 7) interpreting the data. By taking this approach we will be able to develop clusters of themes that facilitate both explanatory and descriptive conclusions directly relevant to our case study approach. Case study analysis calls for early analysis and organisation of data, and coding is one way to do this. We will utilise NVivo for Teams for coding and management of the qualitative data sets [43].

### Health economic evaluation

The payment model has been designed following the net benefit correspondence theorem (NBCT) [17–19, 44] to create continuous quality of care improvement incentives with a conservatively health system budget neutral mechanism; with quality of care incentive payments relative to expected downstream cost savings from improved quality. The NBCT provides a generalised method to enable appropriate incentives for improving health system performance, which, as in hospital applications [17, 19], has robust coverage and comparability conditions to create appropriate accountability and prevent perverse incentives - cost and effect shifting and cream skimming.

Importantly, as quality improvement incentives are conservatively budget neutral or potentially somewhat cost saving to the health system, if the trial finds they are effective in improving quality of care relative to usual care, the mechanisms design provides for strong policy-relevant integration into current practice in a health system budget-constrained environment.

Generalisability, implementation and scalability of any such trial findings from case control analysis will be aided by triangulation with qualitative research and pre-post analysis of impacts in the intervention arm, with particular consideration of barriers and enablers to study findings.

The health economic analysis of trial outcomes will jointly consider the health effects and net costs, including downstream health system (prescribed medication and hospital) costs of a capitation-based GP payment system, where bonus payments are continuous across specified ranges for quality of care measures for each of the activities proposed [17–19, 45].

Analysis with patient level data will include representing joint cost and effect distributions on the cost-effectiveness plane (two strategy comparisons), cost-disutility plane (multiple strategy comparisons) and relevant summary measures. These summary measures include internationally recognised best practice measures of the level of the probability of, and expected gain from, being cost-effective: net benefit and cost-effectiveness acceptability curves for two strategy comparison [46]; expected net loss curves and frontiers for multiple strategy comparisons [17, 18, 45], and expected net loss planes and surfaces with multiple domain of effect comparisons [17, 44].

### Sample size and power

To reduce bias and cross-group contamination, we propose to recruit 36 practices and then randomise practices to intervention and control groups of equal sizes. A total of 1800 patients (50 patients per practice) will then be recruited according to identical inclusion and exclusion criteria. The sample size will allow us to detect a change of 0.2 in the mean score of PCAT relational continuity scale (out of a total maximum of 4.0) with 98% power for adult participants, assuming an intracluster correlation coefficient (ICC) of 0.05 at the practice level. The study is powered to detect a similar change in the other domains of the PCAT [47].

In addition, the trial is powered to detect a 40% reduction in PAHs in the 5 years following the trial in comparison with the 12 months prior to the trial in an adult high-risk population with 80% power, assuming an annual PAH rate of 7.5%, ICC of 0.02, attrition of four practices and 25% attrition of patients from remaining practices. Due to funding and time constraints, it is not possible to adequately power the trial to measure a reduction in PAHs within the study period. Therefore, the study investigators will establish data collection procedures to enable ongoing data collection and analysis for up to 5 years.

### Trial governance

All Chief Investigators are members of the trial steering committee, which meets every 2 weeks. Its remit includes data management. It is independent from the sponsor. Any reports of potential harm from participating general practices or patients will be investigated by the Steering committee and, if appropriate, reported to the ethics committees.

## Discussion

This trial consists of a practice-based intervention implemented in the primary care setting and predominantly using quantitative data collection and analysis. The trial is designed to test the effects of a new funding model that provides financial incentives for continuous quality improvement in general practice, proportional to the expected downstream cost savings. As part of the trial design, nested case studies are planned. These are intended to contextualise the quantitative findings and provide insights into the attitudes and beliefs of patients and practitioners, and their perceptions about the success or otherwise of the intervention.

The development of the intervention was underpinned by several key tenets, namely that:

- high-quality primary health care can reduce the use of secondary care, thus reducing health system costs;
- the design and use of financial models that support high-quality primary care are crucial to the uptake of quality improvement activities and better patient outcomes; and
- financial incentives for quality improvement are more likely to succeed when incentives are continuous and targeted to specific tasks that are closely linked to high-quality care.

Thus, the trial was designed to incentivise GPs to provide enrolled patients with longer consultations, same day access and rapid follow-up after hospitalisation, and to achieve associated quality improvements linked to these tasks. The primary outcome measure of the trial is patient-perceived relational continuity and secondary outcomes include changes in pathology and imaging, total prescriptions and rates of PAHs - all indicators of quality of care.

The design of funding models to support high-quality primary care is crucial to the uptake of quality improvement activities and better patient outcomes [48]. Health economic theory suggests that incentives with GP payments based on fee-for-service can be significantly improved with capitation payments conditional on continual quality improvement [49–54]. Indeed, our review of international financial models for effective primary care indicated that a blend of fee-for-service, capitation and pay-for-performance elements appeared to provide the best balance between improved patient outcomes and productivity. However, the evidence strongly suggested that the incentives were only successful when targeting tasks closely linked to professional standards of high-quality care [48].

The funding model within the trial seeks to provide incentives to encourage:

- access to a preferred provider, longer consultations and structured follow-up after hospitalisation among older and chronically ill general practice patients;
- reduced referrals for specified radiology and pathology services, and overall medicine prescriptions for older and chronically ill general practice patients;
- improved access to same-day care for under 16-year-olds with defined comorbidities; and
- reduced PAHs and emergency department attendances for all enrolled patients.

It is important to note that the absolute incidence of PAHs in the general population is low at around 2.8% per annum, which means that enrolling a non-selected cohort of patients is likely to lead to a null trial with time and funds available. In the 65–74 years age group, the national rate of PAH for 20011/12 was 7,344.8 per 100,000 population (7.3%). For those aged 75 years and over, the rate was 13.4% [55].

Chronic conditions contribute a significant proportion of PAHs, with diabetes complications, COPD, cardiac failure, asthma and angina being most common [56]. Hence, we have focused on high-risk chronic/complex patients aged 18–65 years with the highest-risk ACSCs (COPD, diabetes, angina, ischaemic heart disease, cardiac failure, asthma) expected to have greatest benefit and health system cost saving where the model of care is effective.

The rate of PAHs in the general population under 16 years of age is approximately 2% per annum [55]. Hence, we have targeted recruitment of patients aged less than 16 years to those with an increased risk of hospitalisation. Research from New Zealand has demonstrated a high rate of re-hospitalisation in under 15-year-olds with a defined expert-derived list of child-specific potentially avoidable hospitalisations (approximately 80% of re-hospitalisations up until 15 years of age) [57]. We are therefore using these diagnoses to recruit participants (includes asthma, epilepsy, acute bronchiolitis, pneumonia, croup and dental conditions).

We are using an embedded mixed methods design to incorporate a qualitative component into an outcomes-based randomised controlled trial. This qualitative data will be used to develop understanding of how the intervention is experienced by participants, and to help contextualise and explain the quantitative results. Our analysis aligns with Creswell and Plano Clark’s model of mixing data using embedded mixed method design [58]. At a pragmatic level, we anticipate that by integrating the embedded qualitative component with our quantitative trial data we will better be able to:

- reveal and contextualise relationships between variables and outcomes;
- explore and possibly explain differences in effect size;
- assess the acceptability of the trial model from multiple perspectives; and
- provide insight into discrete, potentially unanticipated impacts of the model on patients and practitioners.

The trial is designed to generate robust, policy-relevant evidence of the impact of a new service model of primary care practice, comprising targeted practice incentives for patient enrolment with a preferred provider, longer consultations, same day access and structured follow-up after hospitalisation, on the quality of primary care and health service utilisation for patients at increased risk of hospitalisation. In addition, the trial will provide evidence on continuous quality improvement incentives which can be compared to block funded pay-for-performance models in the UK [59], the patient-centred medical home model in the United States [60] and the Australian Government ‘Health Care Homes’ trial [31].

### Trial status

The trial is ongoing, with patient recruitment commencing June 1 2018 and completed December 31 2018 and trial completion July 31 2019. The study was in in the intervention and data collection stages at the time of the protocol manuscript submission.

## Data Availability

Not applicable.

## Abbreviations

ACSCs: ambulatory care-sensitive conditions
CMOc: context-mechanism-outcome configurations
COPD: chronic obstructive pulmonary disease
EQ-5D-5 L: five-level version of the EuroQol five-dimensional questionnaire
GP: general practitioner
GPR: general practice registrar (trainee)
IF: intervention facilitator
IRSD: index of relative social disadvantage
MBS: Medicare Benefits Schedule
NBCT: net benefit correspondence theorem
NSW: New South Wales
PAH: potentially avoidable hospitalisation
PBRN: practice-based research network
PBS: Pharmaceutical Benefits Schedule
PCAT: primary care assessment tool
PM: practice manager
PN: practice nurse
QOL: quality of life
RACGP: Royal Australian College of General Practitioners
RCT: randomised controlled trial
SEIFA: socio-economic indexes for areas
