# Correction to: Effectiveness of quality incentive payments in general practice (EQuIP-GP): a study protocol for a cluster-randomised trial of an outcomes-based funding model in Australian general practice to improve patient care

**DOI:** 10.1186/s12913-019-4426-1

**Published:** 2019-08-19

**Authors:** Gregory M. Peterson, Grant Russell, Jan C. Radford, Nick Zwar, Danielle Mazza, Simon Eckermann, Judy Mullan, Marijka J. Batterham, Athena Hammond, Andrew Bonney

**Affiliations:** 1 School of Medicine, University of Tasmania, Hobart and Launceston, Tasmania Australia; 2 Department of General Practice, Monash University, Clayton, Victoria Australia; 3 Faculty of Health Sciences & Medicine, Bond University, Robina, Queensland Australia; 4 Australian Health Services Research Institute, University of Wollongong, Northfields Ave, Wollongong, NSW Australia; 5 School of Medicine, University of Wollongong and Illawarra Health and Medical Research Institute, Northfields Ave, Wollongong, NSW Australia; 6 Statistical Consulting Centre, School of Mathematics and Applied Statistics, University of Wollongong; National Institute for Applied Statistics Research Australia, University of Wollongong; and Illawarra Health and Medical Research Institute, Northfields Ave, Wollongong, NSW Australia; 7 School of Medicine, University of Wollongong, Northfields Ave, Wollongong, NSW Australia

**Correction to: BMC Health Serv Res**

10.1186/s12913-019-4336-2

In the original publication of this article [1], the first name of the 3rd author is wrong.

The correct name should be Jan C. Radford.

The original article has been corrected.
